# Beyond tug-of-war: Iron metabolism in cooperative host–microbe interactions

**DOI:** 10.1371/journal.ppat.1008698

**Published:** 2020-08-12

**Authors:** Grischa Y. Chen, Janelle S. Ayres

**Affiliations:** Molecular and Systems Physiology Lab, Gene Expression Lab, NOMIS Center for Immunobiology and Microbial Pathogenesis, The Salk Institute for Biological Studies, La Jolla, California, United States of America; University of Massachusetts, Worcester, UNITED STATES

## Introduction

Infections induce dramatic rearrangements in host macro- and micronutrient processes [1] and likely reflect host adaptive mechanisms to defend against infection. Traditional models to explain these adaptations suggest antagonistic host–pathogen coevolution. This is probably best exemplified by our current view of host iron metabolism during infection. Iron is an essential micronutrient for all living organisms. Within a host environment, pathogenic microorganisms require host iron to thrive and promote their fitness. In response, mammals have evolved a plethora of strategies that are believed to “starve” invading pathogens of iron, whereas pathogens have evolved ingenious countermeasures to bypass host iron sequestration. However, the role of iron metabolism during infection is far more complex than just a simple “tug-of-war” for nutrients. Here, we highlight recent discoveries demonstrating that iron can promote cooperation in host–microbe interactions, emphasizing alternative ways in which host iron metabolism influences disease outcomes.

## Cooperative defense system in host–microbe interactions

“Antagonistic” interactions between a host and a microbe involve host defense mechanisms that maintain the host’s fitness status while having a negative impact on microbial fitness. By contrast, “cooperation” between a host and a microbe involves host mechanisms that promote host fitness while having a neutral to positive influence on microbial fitness [2]. These mechanisms are encoded by the cooperative defense system that is crucial for an animal’s ability to thrive when interacting with microbes. The cooperative defense system includes disease-tolerance mechanisms that promote host survival during infections by limiting host physiological damage [3–5]. The cooperative defense system also includes antivirulence mechanisms [6], which can tame virulent behavior of microbes within the host niche without altering their ability to replicate. An example is changes in intestinal carbohydrate availability to down-regulate pathogen virulence genes and sustain host health without inhibiting pathogen replication [6, 7]. The distinction between disease tolerance and antivirulence mechanisms is that disease tolerance prevents physiological damage without affecting the microbial pathways that led to disease [8]. Antivirulence mechanisms prevent the induction of such microbial pathways [8].

## Iron metabolism in cooperative host–microbe interactions

The role of iron regulatory pathways in immunity against pathogens has been well studied (reviewed in [9]). However, in recent years iron metabolism has emerged as a critical regulator of cooperative host–microbe interaction. In the following sections, we discuss the role of iron metabolism in cooperative host–microbe interactions through mitochondrial biogenesis, wound healing, detoxification, recycling, and glucose regulation.

### Alternative functions of microbial siderophores on host physiology

Siderophores are molecules that chelate external iron with high affinity and transport iron into microorganisms through dedicated transport systems [10]. Thus, siderophores are essential virulence factors for many microbial pathogens [11]. Enterobacteria produce enterobactin (Ent), a catecholate siderophore, to scavenge iron from the environment [12]. Ent not only promotes growth of pathogens in an iron-deplete host environment but also dampens neutrophil antimicrobial response by chelating neutrophil intracellular labile iron [10, 13]. In response to pathogens, hosts have evolved a litany of tools to sequester iron from pathogens, such as Lipocalin-2, which binds and sequesters Ent [14].

**Fig 1:**
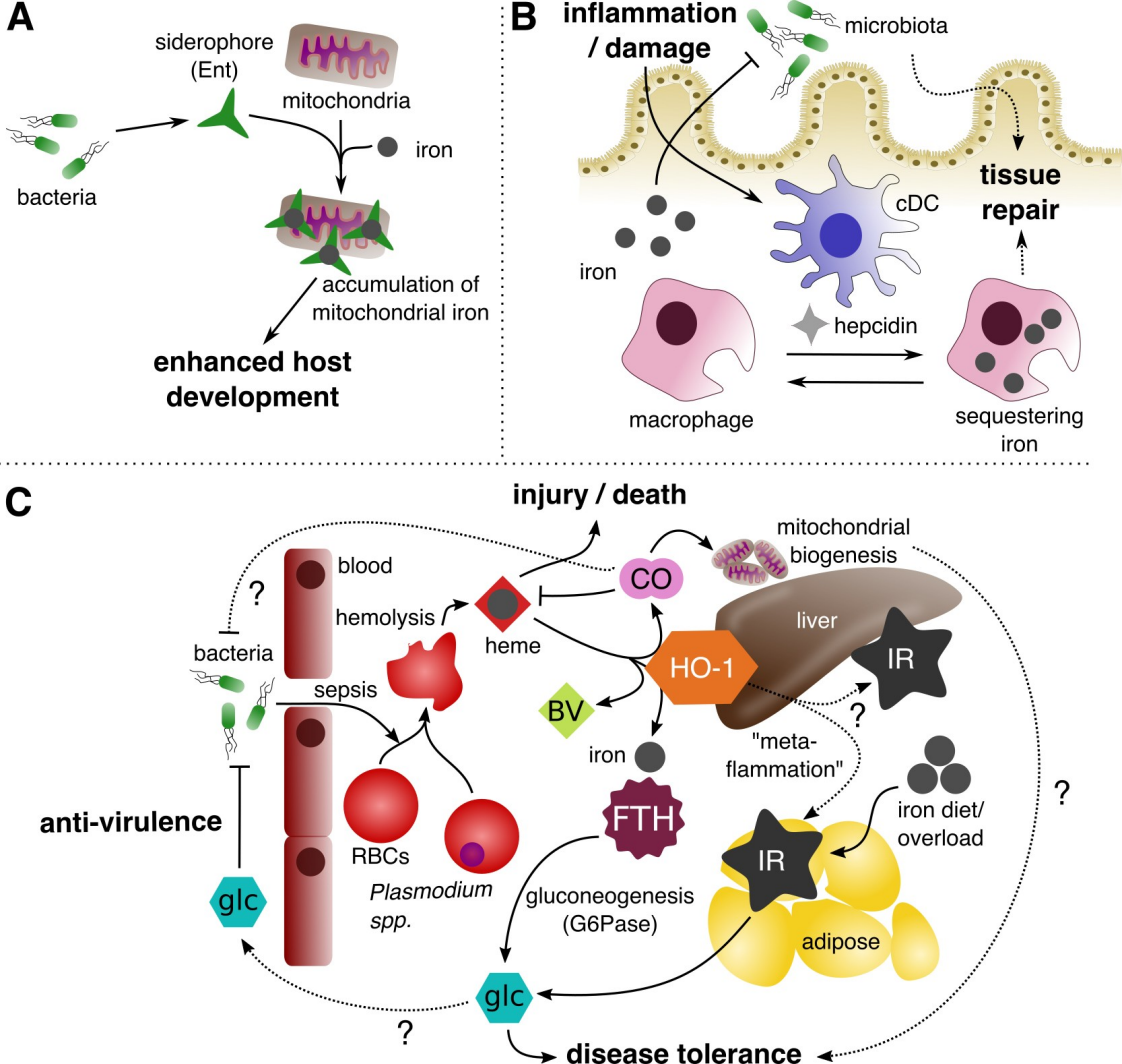
Iron metabolism in cooperative defenses. (A) Alternative functions of bacterial siderophores. Ent, a bacterial siderophore produced by pathogenic and commensal bacteria, chelates host iron. In *C*. *elegans*, Ent was found to promote worm development by binding ATPase components and accumulating iron in the host mitochondria. (B) Hepcidin in wound healing. In response to intestinal damage, cDCs express hepcidin and induce iron sequestration within macrophages and neutrophils. This process is critical for promoting specific microbiota members and facilitates mucosal repair. (C) Host iron metabolism and coregulation of glc metabolism during cooperative defense. During acute sepsis and malaria, reactive heme and iron are released from RBCs and cause cellular injury and death. HO-1 expressed in various cell types degrades reactive heme into iron, CO, and BV. Degradation of heme and production of CO are important antivirulence and disease-tolerance mechanisms to reduce cell injury. Additionally, host HO-1 and FTH through IR and gluconeogenesis, respectively, mediate glc homeostasis. Iron diet also induces insulin resistance through iron overload of the adipose tissue during intestinal infections. The accumulation of glc can provide cooperative defenses by preventing lethal hypoglycemia and attenuating virulence of bacterial pathogens or the microbiota. BV, biliverdin; cDC, conventional dendritic cell; CO, carbon monoxide; Ent, enterobactin; FTH, ferritin; glc, glucose; HO-1, heme oxygenase; IR, insulin resistance; RBC, red blood cell.

In addition to the role of siderophores in antagonistic host–microbe coevolution, siderophores are also critical for interspecies competition between members of the microbiota [15]. These interactions by beneficial microbes can drive exclusion of pathogenic microbes and host protection against infection [16]. Furthermore, siderophores may be important for promoting cooperation in host–microbe interactions. In elegant work, Qi and Han showed that siderophores produced by the intestinal microbiota promote *Caenorhabditis elegans* physiological development [17] (**Fig 1A**). Microbe-derived Ent was found to bind to the alpha subunit of the host mitochondrial ATP synthase, thereby increasing mitochondrial iron levels and host development under both low and high iron conditions. We propose that this work exemplifies iron-mediating cooperative host–microbe interactions because development of the host facilitates replication, nutrient acquisition, and transmission of enterobacteria. Given that other canonical virulence factors for acquiring host iron are present in commensal or beneficial microbes, future work should reassess the role of these systems with the perspective of cooperative host–microbe interactions.

### Hepcidin in wound repair

During acute infections, individuals experience inflammation-dependent hypoferremia [18]. Hepcidin signaling occurs in response to inflammatory signals like interleukin (IL)-6 [19] to prevent duodenal iron absorption and sequester iron within tissues and phagocytes. Hepcidin-deficient mice are highly susceptible to sepsis by *Escherichia coli* and *Vibrio vulnificus*, suggesting that transient hypoferremia is an effective metabolic defense to restrict certain extracellular pathogens [20, 21]. Interestingly, retention of iron within macrophages can promote virulence of intracellular pathogens such as *Salmonella enterica*, *Burkholderia pseudomallei*, *Chlamydia* spp., and *Legionella pneumophila* [22–24].

In addition to hepatocytes, myeloid cell types are also a source of hepcidin production [25]. In response to intestinal mucosal insult, Bessman and colleagues show that type 2 conventional dendritic cell (cDC)-derived hepcidin induces sequestration of iron in macrophages and neutrophils (**Fig 1B**). Regulation of iron levels was necessary for proper microbiome composition and mucosal repair because cDC-specific hepcidin-deficient mice were slower to recover following intestinal damage [26]. Therefore, unlike hepatocyte-derived hepcidin required for systemic infections, cDC-derived hepcidin promotes intestinal homeostasis. In the future, it will be interesting to examine how this repair system functions during invasion by enteric pathogens.

### Heme detoxification and recycling

During infections, microbial and host-derived toxic compounds can be generated that cause tissue damage. Detoxification mechanisms serve as antivirulence mechanisms to promote cooperative defenses by preventing damage to the host without affecting pathogen burdens [8]. During malaria and acute septicemia, extensive hemolysis leads to release of reactive heme molecules into the bloodstream. Free heme molecules can dampen immune defenses by disrupting immune cell function [27, 28] and also cause liver and kidney damage that ultimately progresses to failure of these organs [29, 30].

Research into heme recycling and detoxification demonstrates the importance of these pathways for cooperative defenses (Fig 1C). Heme degradation and recycling are mediated through heme oxygenase 1 (HO-1), which is highly expressed in phagocytes and degrades heme into biliverdin, carbon monoxide (CO), and iron [31]. Mice defective in HO-1 display severe survival defects when infected with *Plasmodium* or subjected to intestinal perforation leading to systemic bacterial infection [29, 30]. This increased sensitivity to infection is associated with no difference in parasite load or bacteremia, suggesting this enzyme serves as a cooperative defense mechanism instead of promoting resistance defenses of the host. Using genetic knockouts of HO-1 gene, Seixas and colleagues show that HO-1 mitigates hepatocyte injury resultant of free heme released during *Plasmodium* infection [30]. Subsequent studies suggest that CO is a critical component of HO-1’s protective functions in the host. One possibility is that CO inhibits components of the respiratory chain in bacteria [32], thereby modulating microbial metabolism/virulence in vivo. Additionally degradation of heme and CO release works 2-fold by halting heme release from hemoglobin and disruption of the blood brain barrier, thereby reducing cases of cerebral malaria [33]. The HO-1/CO pathway may also prevent organ failure by promoting mitochondrial biogenesis during sepsis [34]. Given the danger of heme toxicity during infection, the presence of multiple complementary pathways supporting host survival is no surprise. Future studies may uncover ways in which heme and its degradation products modulate microbial metabolism and virulence during infection.

### Cross talk between iron and glucose homeostasis

Recent dietary and metabolic studies in animals and humans link iron metabolism to glucose homeostasis at many levels (**Fig 1C**). Weis and colleagues established that the ferritin, the intracellular iron storage protein, regulates hepatic gluconeogenesis and is critical for host survival during polymicrobial sepsis [35]. Knockout of ferritin specifically in hepatocytes increased susceptibility to polymicrobial sepsis without a change in bacterial burdens. Unlike control mice, ferritin mice were unable to restore physiological levels of glucose later during sepsis, suggesting that ferritin is required to reverse sickness-induced, lethal hypoglycemia. Another recent study reveals that HO-1 drives chronic inflammation and insulin resistance in mice, termed “metaflammation.” Both hepatic and macrophage-specific knockouts of HO-1 display improved insulin sensitivities, whereas overexpression of HO-1 drives insulin resistance [36]. These studies highlight the intersection between glucose and iron metabolism in host–pathogen interactions. In future studies, it will be important to consider the coregulation of iron and glucose metabolism and their impact on disease outcomes.

Sanchez and colleagues found that administration of dietary iron and transient insulin resistance promotes survival of mice given oral doses of the diarrheal pathogen *Citrobacter rodentium* [6]. Dietary iron causes iron overload and insulin resistance in white adipose tissue (WAT) [37]. Consistent with this, administration of dietary iron during *C*. *rodentium* infection caused increased tissue iron levels in the WAT and insulin resistance. The insulin resistance caused a reduction in the amount of glucose absorbed from the intestine into the bloodstream, increasing the amount of glucose available to the pathogen to metabolize, which suppressed the virulence program of *C*. *rodentium* [6]. The work by Sanchez and colleagues may represent a general mechanism employed by the host to feed its gut microbes during disease states [6]. Together, this work shows a broader role for iron tissue sequestration and insulin resistance in regulating intestinal microbe virulence and may be an important host response during sickness by taming the virulence of the microbiome when nutrients are scarce.

## Concluding remarks and future perspectives

An entire field, called nutritional immunity, has focused on iron and other micronutrients in antagonism between host and invading microorganisms [38]. Some interesting examples of the evolutionary arms race are heritable hemochromatosis (HH) and sickle cell anemia in humans, which result in iron storage disorders but also confer resistance to human diseases such as tuberculosis, typhoid fever, and malaria [39, 40]. In the same vein, we posit that cooperative defense systems regulating iron metabolism have also evolved to promote health and/or attenuate infectious pathogens. Furthermore, the human gut microbiota is known to play an important role in nutritional immunity by competing with pathogenic microorganisms through competition for iron and other nutrients (termed colonization resistance) [41]. Only recently have people begun appreciating the importance of the microbiota in human health and noncommunicable diseases. Almost all human-associated microorganisms, with a few exceptions, require iron to exist in the host; therefore, it will be interesting to see how host iron metabolism modulates microbiota function, as well as how microbiota utilization of iron modulates host health. In nature, it has been shown that iron can be traded for nutrients during mutualistic interactions [42]. It remains to be seen whether analogous interactions have evolved between humans and the gut microbiota to promote cooperative host–microbe interactions. Uncovering other mechanisms of cooperative defenses may have tremendous impact on how we approach infectious disease treatments to promote host fitness and not select for adverse traits in pathogens (i.e., antibiotic resistance) [5].
